# TCR Repertoire Analysis Reveals Mobilization of Novel CD8^+^ T Cell Clones Into the Cancer-Immunity Cycle Following Anti-CD4 Antibody Administration

**DOI:** 10.3389/fimmu.2018.03185

**Published:** 2019-01-24

**Authors:** Hiroyasu Aoki, Satoshi Ueha, Shigeyuki Shichino, Haru Ogiwara, Shin-ichi Hashimoto, Kazuhiro Kakimi, Satoru Ito, Kouji Matsushima

**Affiliations:** ^1^Department of Molecular Preventive Medicine, Graduate School of Medicine, The University of Tokyo, Tokyo, Japan; ^2^Division of Molecular Regulation of Inflammatory and Immune Diseases, Research Institute for Biomedical Sciences, Tokyo University of Science, Noda, Japan; ^3^Division of Nephrology, Department of Laboratory Medicine, Kanazawa University, Kanazawa, Japan; ^4^Department of Immunotherapeutics, The University of Tokyo Hospital, Tokyo, Japan; ^5^IDAC Theranostics, Inc., Tokyo, Japan

**Keywords:** anti-CD4 mAb, immune checkpoint inhibitor, TCR repertoire, Cancer-Immunity Cycle, Inter-Organ Clone Tracking

## Abstract

Depletion of CD4^+^ cells using an anti-CD4 monoclonal antibody (anti-CD4 mAb) induces the expansion of tumor-reactive CD8^+^ T cells and strong antitumor effects in several murine tumor models. However, it is not known whether the anti-CD4 mAb treatment activates a particular or a broad spectrum of tumor-reactive CD8^+^ T cell clones. To investigate the changes in the TCR repertoire induced by the anti-CD4 mAb treatment, we performed unbiased high-throughput TCR sequencing in a B16F10 mouse subcutaneous melanoma model. By Inter-Organ Clone Tracking analysis, we demonstrated that anti-CD4 mAb treatment increased the diversity and combined frequency of CD8^+^ T cell clones that overlapped among the tumor, draining lymph node (dLN), and peripheral blood repertoires. Interestingly, the anti-CD4 mAb treatment-induced expansion of overlapping clones occurred mainly in the dLN rather than in the tumor. Overall, the Inter-Organ Clone Tracking analysis revealed that anti-CD4 mAb treatment enhances the mobilization of a wide variety of tumor-reactive CD8^+^ T cell clones into the Cancer-Immunity Cycle and thus induces a robust antitumor immune response in mice.

## Introduction

Immune checkpoint inhibitor treatments such as blocking antibodies against cytotoxic T-lymphocyte associated protein 4 (CTLA-4) and programmed death receptor 1 (PD-1) have produced remarkable clinical effects in several kinds of malignancies (1–3). However, the rate of objective responses to anti-CTLA-4 or anti-PD-1 treatment is still low, and hence, the development of additional therapeutic options for immune checkpoint inhibitor-refractory solid cancer has become an urgent requirement.

CD4^+^ T cells play important roles in both humoral and cellular immune responses to pathogens. However, we and other groups have reported that depletion of CD4^+^ cells using a depleting monoclonal antibody results in strong antitumor effects in murine tumor models (4–6) and induces robust antitumor effects when administered synergistically with immune checkpoint inhibitors including anti-PD-1/PD-L1 antibody and anti-CTLA-4 antibody (7). The antitumor effects induced by anti-CD4 depleting monoclonal antibodies (anti-CD4 mAb) were found to be mediated by CD8^+^ cytotoxic T lymphocytes (CTLs), which increased in the draining lymph node (dLN) and tumor after anti-CD4 mAb treatment (7). The enhancement of CD8^+^ T cell responses can be explained by the depletion of several immunosuppressive CD4^+^ cells including forkhead box P3 (Foxp3)^+^ CD4^+^ regulatory T cells (Tregs) . However, it is not known whether the anti-CD4 mAb treatment expands a part of the highly activated tumor-specific CTL clones or mobilizes a wide variety of tumor-reactive CD8^+^ T cell clones into the antitumor CTL response.

The TCR repertoire, a collection of T cell clones generated by V(D)J recombination in the thymus, would be a novel axis for monitoring T cell responses, because T cell clones specific to particular antigens seem to be the elementary unit of adaptive T cell responses (8). In the field of cancer immunology, several clinical studies have already investigated the relationship between the antitumor effect following immunotherapy and features of the TCR repertoire of peripheral blood lymphocytes (PBL) (9–13) or tumor infiltrating leukocytes (TILs) (14, 15). In anti-CTLA-4 treatment, the diversity of TCR repertoire in PBL increased after treatment (10), and in anti-PD-1 treatment, patients with improved survival showed high pre-treatment TCR repertoire diversity and greater post-treatment expansion of tumor-associated clones in PBL (11). TCR repertoire analysis of TILs revealed that responders to anti-PD-1 treatment increase clonality of the TIL repertoire following treatment (14). In addition, responders to anti-CTLA-4 and anti-PD-1 sequential immune checkpoint blockade showed a higher clonality after anti-CTLA-4 treatment (15). However, despite the advantage in the availability of PBL, the relationship between PBL repertoire and TIL repertoire or antitumor T cell response exerted in the tumor remains unclear.

In the series of steps generating antitumor immunity (i.e., the Cancer-Immunity Cycle), tumor-reactive T cells are primed and activated in the dLN and trafficked via blood circulation to the tumor, where they exercise antitumor responses (16). Therefore, to assess the CD8^+^ T cell clone responses in the context of the Cancer-Immunity Cycle (i.e., the expansion site and inter-tissue migration of tumor-infiltrating clones), the T cell repertoire in the dLN, PBL, and tumor must be analyzed simultaneously. We hypothesized that overlapping CD8^+^ T cell clones between the dLN, PBL, and tumor would reflect the spatiotemporal response of T cell clones in the Cancer-Immunity Cycle. To this end, we investigated the changes in the dLN, PBL, and tumor CD8^+^ T cell repertoires following anti-CD4 mAb treatment using unbiased high-throughput TCR sequencing (TCR-seq) in a B16F10 mouse subcutaneous melanoma model.

## Materials and Methods

### Mice and Cell Lines

Six-week-old female C57BL/6 mice (CD90.2) were purchased from Japan SLC (Hamamatsu, Japan). CD90.1 congenic mice with gp100 melanoma antigen-specific Pmel-1 TCR transgene were purchased from The Jackson Laboratory (Bar Harbor, ME). B16F10 is a gp100^+^ spontaneous murine melanoma cell line, kindly provided by Dr. N. Restifo (National Cancer Institute, MD).

### Tumor Therapy

B16F10 cells (5 × 10^5^ cells /mouse) were inoculated subcutaneously (s.c.) into the right flanks of C57BL/6 mice, which were adoptively transferred with 2 × 10^4^ cells of Pmel-1 TCR transgenic CD8^+^ T cells (CD90.1, TCRVβ13) 10 days previously. Anti-CD4 mAb (clone GK1.5, BioXcell, West Lebanon, NH) was injected intraperitoneally (i.p.) at a dose of 200 μg per mouse on days 5 and 9 after tumor inoculation. In this treatment protocol, the frequency of CD4^+^ T cells decreases from 50- to 100-fold at least from days 7 to 14 following tumor inoculation (7). All animal experiments were conducted in accordance with institutional guidelines with the approval of the Animal Care and Use Committee of the University of Tokyo.

### Flow Cytometry and Cell Sorting

Single-cell suspensions from the dLN, PBL, and tumor were prepared by enzymatic or mechanical dissociation of tissues with or without subsequent density separation on day 14, as described previously (7). Intravascular leukocytes were stained by intravenous injection of fluorophore-conjugated anti-CD45 mAb (3 μg/mouse, clone 30-F11) at 3 min before sacrifice (17). The cellular density of the suspensions was determined using Flow-Count fluorospheres (Beckman Coulter, San Diego, CA) and a Gallios flow cytometer. Cells were pretreated with Fc Block (anti-mouse CD16/CD32 mAb; clone 2.4G2, BioXcell), and then stained with a mix of fluorophore-conjugated anti-mouse mAbs: anti-CD90.1 (clone OX-7), anti-CD4 (clone RM4-4), anti-CD11b (clone M1/70), anti-B220 (clone RA3-6B2), anti-CD44 (clone IM7), anti-CD279 (clone RMP1-30), and anti-CD8a (clone 53-6.7). Purified or fluorochrome-conjugated mAbs were purchased from BD Biosciences (San Jose, CA) or BioLegend (San Diego, CA). For the purification of CD8^+^ T cells, CD4, and lineage (CD11b, CD19, NK1.1, and Ter119) positive cells were depleted using an AutoMACS Separator (Miltenyi Biotec, Bergisch Gladbach, Germany), and CD8^+^ T cells or CD8^+^ CD44^hi^ T cells were then sorted on FACS Aria II (BD Biosciences). The purity of sorted cells was always over 95%. Data were analyzed using FlowJo software (version 10.3; FlowJo, LLC, Ashland, OR).

### High-Throughput Sequencing of the TCR Repertoire in the dLN, PBL, and Tumors

TCR-seq libraries for next generation sequencing (NGS) were prepared from the mRNA of sorted T cell samples. Details of the modified procedure have been described in the Supplementary Material. In short, total mRNA was converted to cDNA using reverse transcriptase, and universal sequences for unbiased PCR amplification were added to the 5′ end by poly-A tailing with the terminal deoxynucleotidyl transferase reaction and subsequent second strand synthesis using a universal primer. The TCR locus was amplified by nested PCR with the universal primer and the TCR constant region-specific primers. Amplified TCR libraries were then fragmented enzymatically and sequencing adaptors and barcodes were added to the TCR libraries using ligation and subsequent PCR. Final TCR libraries with 200–300 base pairs were sequenced using an Ion Proton next generation sequencer (Thermo Fisher Scientific). The raw data from these experiments have been deposited at the NCBI GEO; accession GSE115425. The sequences of primers and adapters are shown in Supplementary Table 1.

### Alignment and Assembly of TCR-seq Data

Sequencing data were processed by MiXCR-1.8.2 (18). Adapter sequences and low-quality reads were trimmed using cutadapt-v1.11 (19) and PRINSEQ-0.20.4 (20). Filtered reads were aligned to ImMunoGeneTics (IMGT) reference mouse TCR V/D/J sequences with the following parameters: -OvParameters.geneFeatureToAlign = VTranscript – OvjAlignmentOrder = JThenV, and identical sequences were then assembled and grouped into clones with PCR and sequencing error correlations using the following parameters: -ObadQualityThreshold = 10.

### Analysis of the TCR Repertoire of CD8^+^ T Cells

The list of final clones generated by MiXCR was analyzed using VDJtools (ver 1.1.1.) (21). Non-functional clones containing a stop codon or frameshift in their receptor sequence were eliminated by the “*FilterNonFunctional*” command in VDJtools. Then, the sequencing coverage of samples, which was defined as the ratio of total reads to the starting number of T cells, was normalized to × 9 in TCRβ and × 5 in TCRα using the “*DownSample*” command in VDJtools. The frequency of Pmel-1 TCR was identified by its CDR3 nucleotide sequence and compared with the frequency of Pmel-1 cells detected by flow cytometry. The Variable (V) and Joining (J) segment of TCRs were represented in IMGT gene nomenclature. The coverage-normalized data have been deposited at the NCBI GEO; accession GSE115425.

### Statistical Analysis

Data are presented as mean ± SE of 5 mice per group, except for PBL of aCD4 group: *n* = 3. Unless otherwise stated, the T cell clones were determined as TCR reads with the same TCR Variable (V) segment, Joining (J) segment, and CDR3 nucleotide sequence. The clonality of the TCR repertoire was calculated as 1-Pielou index, which was calculated using the formula $1+\;\sum_{i=1}^{n}\left(p_{i}*\ln(p_{i}))/\ln(\text{n}\right)$, where *p*_*i*_ is the frequency of clone *i* for a sample with *n* unique clones. Of note, this metric is normalized to the number of unique clones and ranges from 0 to 1. The TCR repertoire diversity was determined as the number of clones whose frequency was higher than 0.01%. Statistical analyses were performed using GraphPad Prism (ver7) software (GraphPad Software, La Jolla, CA, USA). The Pearson product-moment correlation coefficient was calculated to determine the accuracy and reproducibility of our TCR-seq method. For comparisons between the means of two variables, we used two-sided unpaired Student's *t*-tests, with ^*^, ^**^, and ^***^ indicating *P* < 0.05, 0.01, and 0.001, respectively.

## Results

### Unbiased TCR Sequencing of the CD8^+^ T Cell Repertoire in Individual Tumor-Bearing Mice

**Figure 1:**
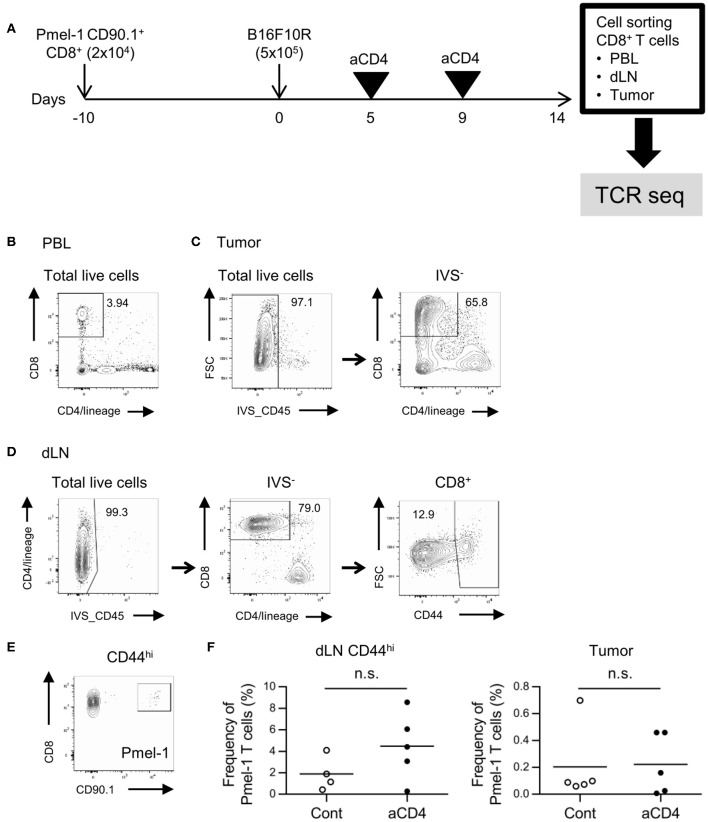
Gating strategy for CD8^+^ T cells in the dLN, PBL, and tumor. **(A)** Experimental procedure. Melanoma antigen-specific TCR (TCRVα1Vβ13; Pmel-1) expressing CD8^+^ T cells with the CD90.1 congenic marker was adoptively transferred 10 days prior to B16F10 tumor inoculation into C57BL/6 mice (CD90.2). Tumor-bearing mice were injected i.p. with anti-CD4 mAb on days 5 and 9, and CD8^+^ T cells in the dLN, PBL, and tumor were isolated using cell sorters. **(B–D)** Flow cytometry plots showing CD8^+^ T cells in the PBL **(B)**, tumor **(C)**, and dLN CD44^hi^ population **(D)**. Numbers in flow-cytometry plots indicate frequencies within parental populations **(B–D)**. A similar gating strategy was used for CD8^+^ T cell isolation using cell sorters. **(E)** Flow cytometry plots showing the CD8^+^ Pmel-1 T cells in the dLN. A similar gating strategy was used in PBL and tumor. **(F)** Frequency of Pmel-1 T cells in dLN CD44^hi^ (left) and tumor (right) by flow cytometry. Two-sided unpaired Student's *t*-tests (*n* = 5, except for dLN CD44^hi^ of aCD4: *n* = 4).

To investigate the effect of anti-CD4 mAb treatment on the TCR repertoire, we adopted the B16F10 mouse melanoma model (Figure 1A). C57BL/6 mice were adoptively transferred with Pmel-1 CD8^+^ T cells, which express melanoma antigen-specific TCR, 10 days before inoculation with B16F10 tumors. Tumor-bearing mice were left untreated (control) or injected i.p. with anti-CD4 mAb on days 5 and 9 after tumor inoculation (aCD4). On day 14, the unfractionated CD8^+^ T cells in the blood and tumor, and CD44^hi^ CD8^+^ T cells in the dLN were purified for the TCR repertoire analysis (Figures 1B–D). Enrichment of the CD44^hi^ effector/memory population excluded the antigen inexperienced naïve CD8^+^ T cell population that predominates in the dLN. Flow cytometry analyses revealed the successful induction of B16 reactive Pmel-1 CD8^+^ T cells following aCD4 mAb treatment in the dLN CD44^hi^; in the aCD4 group, the frequency of Pmel-1 T cells tended to increase in dLN CD44^hi^ (control; 1.9 ± 0.8%, aCD4; 4.5 ± 1.4%, *P* = 0.18), however, the frequency did not change in the tumor (control; 0.20 ± 0.12%, aCD4; 0.22 ± 0.10%, *P* = 0.91) (Figures 1E,F).

We next prepared unbiased TCR-seq libraries for NGS from the mRNA of sorted CD8^+^ T cell samples (Supplementary Figures 1A,B, Supplementary Table 1) and then the resulting TCR libraries were sequenced using the Ion Proton next generation sequencer with a coverage > 5 × (TCRα) or > 9 × (TCRβ) (Supplementary Tables 2, 3). The accuracy of the sequencing result was certified by Pearson's correlation of the frequency of Pmel-1 cells in NGS reads and flow cytometry. Reproducibility of the sequencing platform was also certified by Pearson's correlation of the frequency of overlapping clones between technical replicate samples (Supplementary Figures 2A–C).

### The CD8^+^ T Cell Repertoire in Tumors Exhibited a Distinctive Structure Compared to dLN and PBL Repertoires

**Figure 2:**
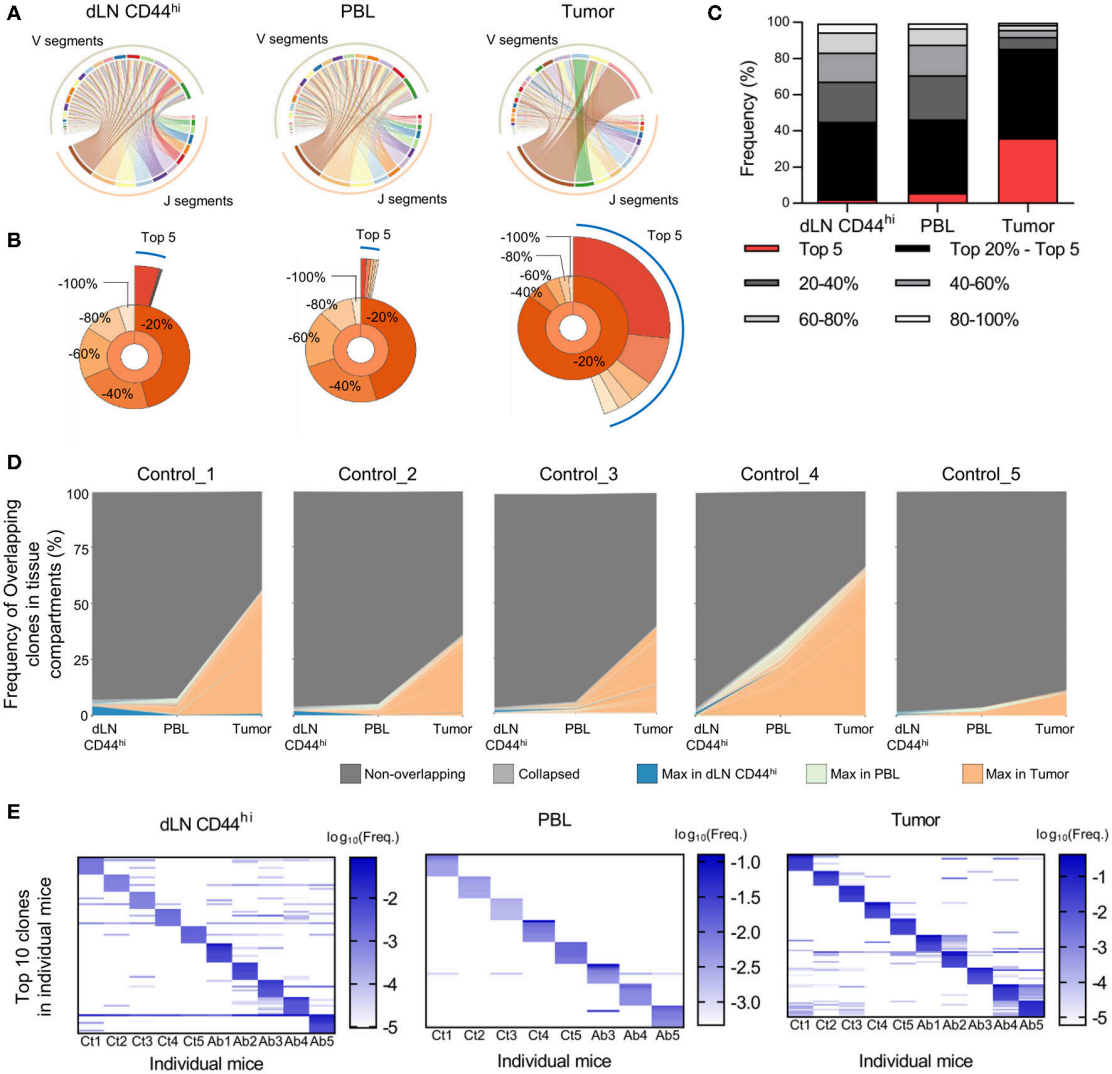
Differences in TCR repertoires between the dLN, PBL, and tumor in tumor-bearing mice. Mice bearing B16F10 tumors were analyzed on day 14 after tumor inoculation. **(A)** V/J segment usage plots of the dLN CD44^hi^, PBL, and tumor repertoires of a control mouse. Ribbons connecting the V and J segments are scaled by the corresponding V/J pair frequency. **(B)** Quantile statistics of the dLN CD44^hi^, PBL, and tumor in a control mouse. The second layer of the pie chart displays the combined frequency of the top 20, 20–40, 40–60, 60–80, and 80–100% clones. The third layer displays the individual frequency of the top 5 clones. **(A,B)** are figures depicting data from the same mouse. **(C)** Average of quantile statistics in control mice (*n* = 5). **(D)** IOCT analysis in individual control mice. The top 100 overlapping clones in at least two compartments are colored by the tissue compartment in which their frequency was the highest, whereas the clones whose frequencies were below the top 100 are shown as light gray and labeled “Collapsed” in the figure legend. **(E)** Variability of TCR repertoire between individual mice in the dLN CD44^hi^, PBL, and tumor. The top 10 clones of individual mice were plotted on the heat map. Each column and row represent the individual mice and clones, respectively. Colors represent the log_10_ scaled frequency of clones in each mouse as indicated by a color scale. Undetected clones are colored white. **(A–C)** present the data of control group. In **(E)** T cell clones were determined as the TCR reads with the same TCR V segment, J segment, and CDR3 amino acid sequence. All figures present the data for TCRβ.

We next investigated the differences in TCR repertoires among the dLN, PBL, and tumor in individual tumor-bearing mice. We first compared the Variable-Joining (V/J) segment usage of TCRβ of CD8^+^ T cells among the dLN CD44^hi^, PBL, and tumor (Figure 2A). In the tumor repertoire, ribbons connecting the V and J segments were thicker than those in the dLN CD44^hi^ or PBL, indicating that the repertoire in the tumor had a more skewed V/J segment usage than the other two compartments. Similar and clearer trend was observed for the V/J segment usage of TCRα (Supplementary Figure 3A). Second, we compared the frequency of the top 5 and top 20% clones in CD8^+^ T cell repertoire among the dLN CD44^hi^, PBL and tumor (Quantile stats, Figure 2B). In the control group, the frequency of the top 5 and top 20% clones in the tumor was higher than those in the PBL and the dLN CD44^hi^ (Figure 2C). TCRα repertoire also showed similar tendencies (Supplementary Figures 3B,C). These observations suggested that oligoclonal expansion of CD8^+^ T cells occurred in the tumor.

### A Small Fraction of the T Cell Repertoire in dLN or PBL Was Enriched in the Tumor

To follow the spatiotemporal response of CD8^+^ T cells in the Cancer-Immunity Cycle with respect to the TCR repertoire, we performed IOCT analysis on TCRβ repertoire by plotting the frequencies of overlapping clones in each tissue compartment (Figure 2D). On an average, 144 ± 62 clones were overlapped in at least two compartments of the control group. The combined frequency of overlapping clones was highest in the tumor (41.9 ± 21.1%), and was relatively low in the dLN CD44^hi^ (3.6 ± 2.0%) and PBL (10.8 ± 12.1%). TCRα repertoire also showed similar tendencies (Supplementary Figure 3D). These results suggest that only a small fraction of clones in the dLN CD44^hi^ or PBL accumulated in the tumor. In addition, we examined the variability of TCR repertoire between individual mice. To this end, we compared the frequency of top 10 TCRβ clones of the dLN CD44^hi^, PBL, and tumor between individual mice (Figure 2E). We observed that many of top 10 clones in one individual did not exist or existed at a very low frequency in the others, particularly in the PBL and tumor repertoire. Moreover, the combined frequency of public clones (i.e., clones shared between at least two mice) in each repertoire was variable between mice in the tumor (9.0–88.0%, mean = 48.2%), and was relatively small in dLN CD44^hi^ and PBL (dLN CD44^hi^; 18.0–31.9%, mean = 23.1%, PBL; 2.0–5.4%, mean = 3.3%). Therefore, the TCR repertoire should be analyzed for each individual and the repertoire of the same treatment group or tissue compartment should not be pooled. However, this tendency was less clear in the TCRα repertoire in the dLN CD44^hi^ and tumor, suggesting the presence of public clones in TCRα repertoire of C57BL/6 mice inoculated with B16F10 melanoma (Supplementary Figure 3E).

### Anti-CD4 mAb Treatment Increases the High Frequency Clones in dLN

**Figure 3:**
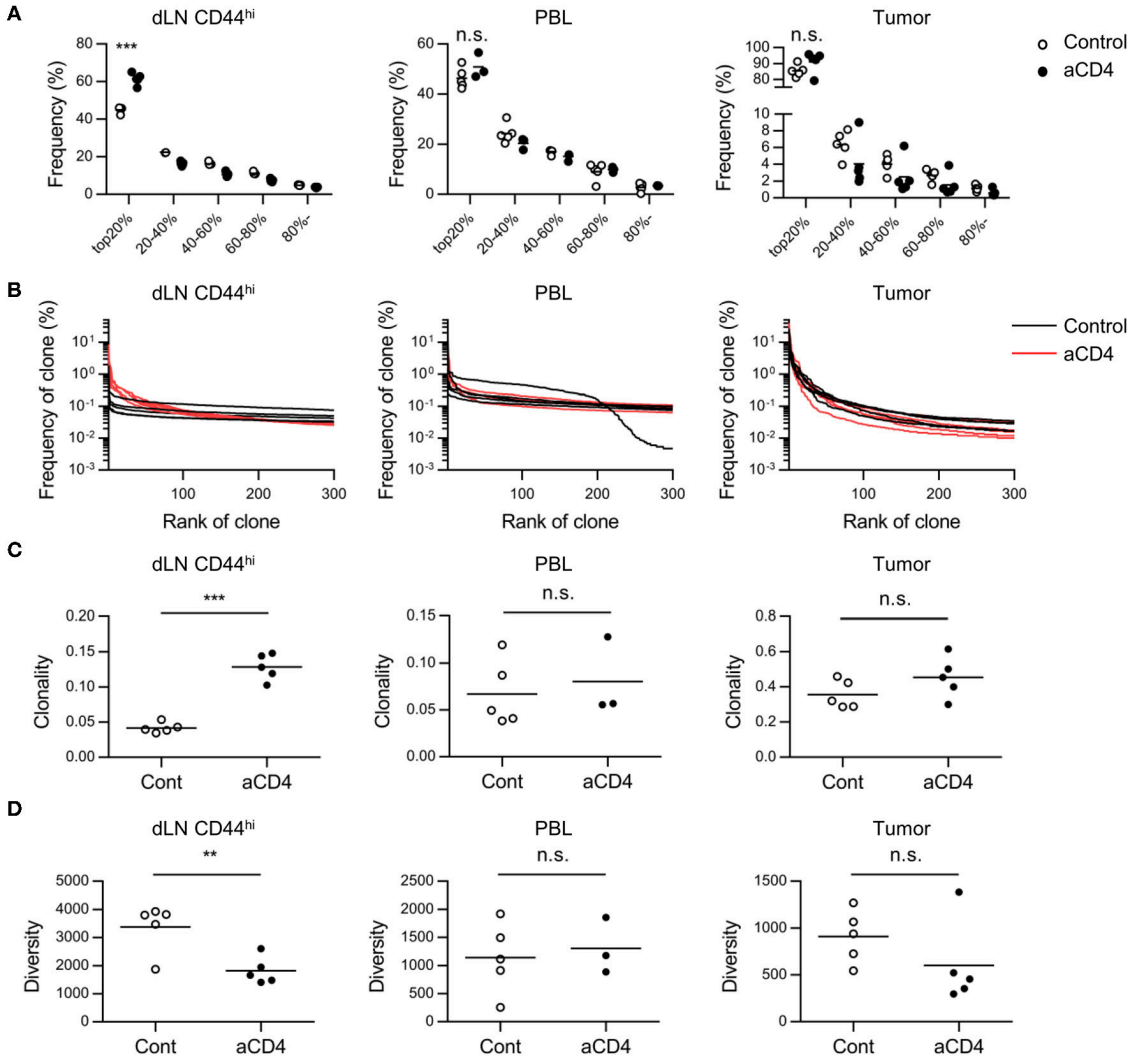
Anti-CD4 mAb treatment triggered the expansion of high frequency clones in dLN. Mice bearing B16F10 tumors were injected i.p. with anti-CD4 mAb on days 5 and 9, and were analyzed on day 14 after tumor inoculation. **(A)** Quantile statistics of the dLN CD44^hi^, PBL, and tumor repertoire. **(B)** Distribution plots of the dLN CD44^hi^, PBL, and tumor repertoire. The x-axis represents each clone in the descending order of frequency, and y-axis represents the frequency of each clone in the dLN CD44^hi^, PBL, and tumor repertoires. **(C,D)** Clonality **(C)** and diversity **(D)** of the dLN CD44^hi^, PBL and tumor repertoire. Clonality was calculated as 1-Pielou index (method). Diversity was defined as the number of clones with a frequency of >0.01%. All figures present the data for TCRβ. Two-sided unpaired Student's *t*-tests (*n* = 5, except for PBL of aCD4: *n* = 3). ^**^P < 0.01; ^***^P < 0.001.

In order to understand the changes in the CD8^+^ T cell repertoire following anti-CD4 mAb treatment, we compared the TCRβ repertoire of CD8^+^ T cells in each compartment alone between the control and aCD4 groups. In the aCD4 group, the frequency of the top 20% clones among dLN CD44^hi^ T cells increased significantly (Figure 3A; control; 44.9 ± 0.7%, aCD4; 61.5 ± 1.4%, *P* < 0.0001). The frequency distribution of the top 300 clones is depicted to analyze the repertoire structure more precisely (Distribution plot, Figure 3B). In the dLN CD44^hi^, the aCD4 group exhibited an upward shift of the distribution plot, which suggested the increase of high frequency clones in the dLN of anti-CD4 mAb treated mice. Then, to clarify the changes in repertoire structure following the anti-CD4 mAb treatment, the clonality and diversity of the repertoire were compared in each compartment (Figures 3C,D). Clonality was calculated as the 1-Pielou index, which is inversely proportional to diversity, i.e., a higher clonality index indicates a more uneven repertoire structure generated by the increase of selected clones. In dLN CD44^hi^, the aCD4 mice exhibited higher clonality (control; 0.042 ± 0.003, aCD4; 0.129 ± 0.008, *P* < 0.0001) and lower diversity (control; 3,377 ± 384, aCD4; 1817 ± 217, *P* = 0.0076), which matched the changes in the distribution plots of the aCD4 mice. The TCRα repertoire showed similar tendencies, and these anti-CD4 mAb treatment-induced changes in repertoire structure were detected more clearly in dLN CD44^hi^ rather than in the whole dLN (Supplementary Figures 4A–D). In contrast to the dLN repertoire, the PBL, and tumor repertoires of the aCD4 group did not show such clear tendencies in the frequency of the top 20% clones, distribution plots, clonality, and diversity (Figures 3A–D and Supplementary Figures 4A–D).

### Anti-CD4 mAb Treatment Increased Overlapping Clones Between the Tumor and dLN

**Figure 4:**
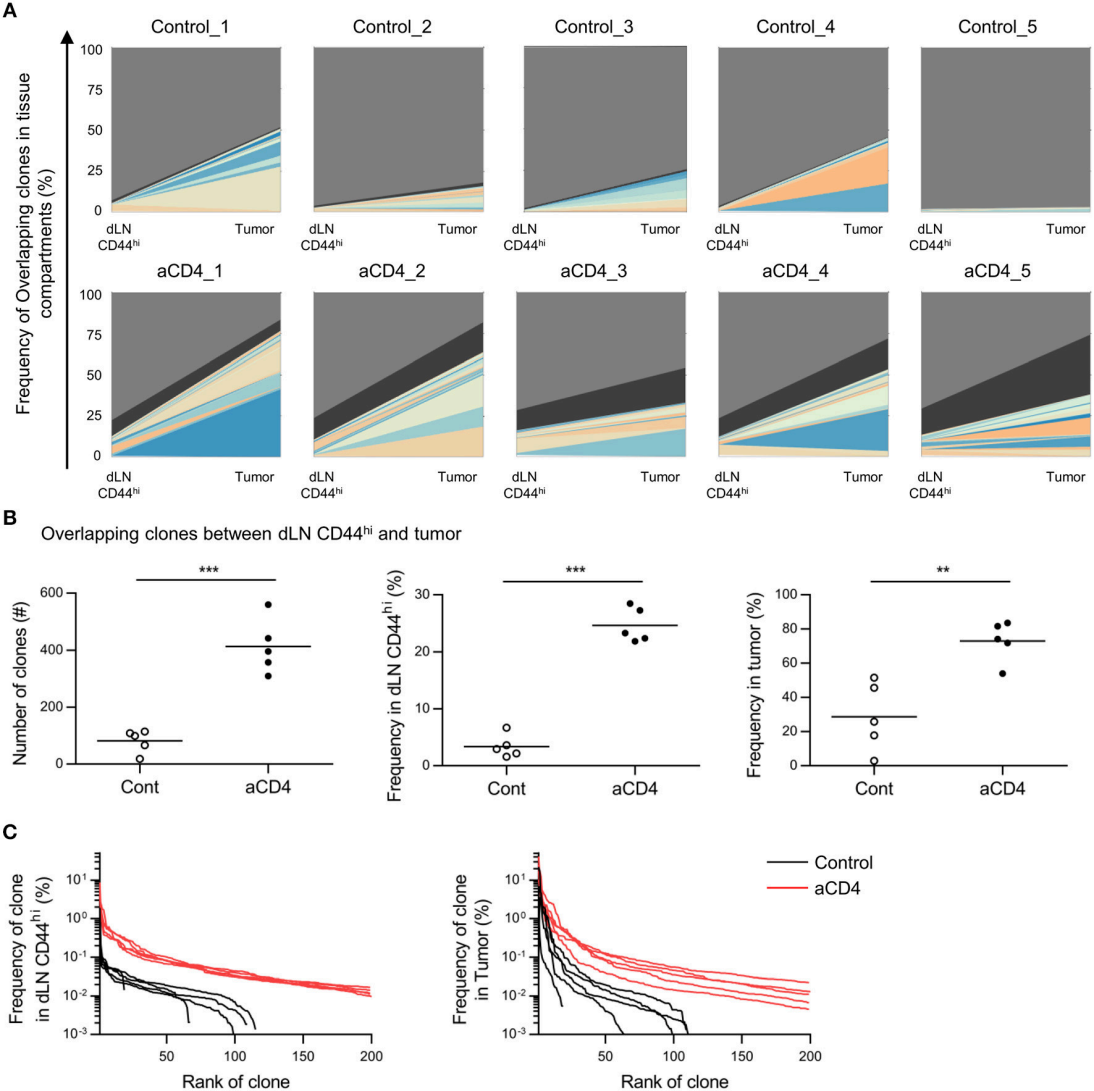
Anti-CD4 mAb treatment increased the dLN-tumor overlap. Mice bearing B16F10 tumors were injected i.p. with anti-CD4 mAb on days 5 and 9, and were analyzed on day 14 after tumor inoculation. **(A)** Overlap analysis between the dLN CD44^hi^ and tumor in individual mice. Plots show the top 20 clones overlapping between dLN CD44^hi^ and tumor, as well as the clones whose frequency were below the top 20 (black) and non-overlapping (gray) clones. **(B)** Overlapping clones between the dLN CD44^hi^ and tumor. The total number (left), combined frequency in the dLN CD44^hi^ (middle), and combined frequency in the tumor (right) were compared. **(C)** Distribution plots of overlapping clones between the dLN CD44^hi^ and tumor. The x-axis represents each overlapping clone in the descending order of frequency, and the y-axis represents the frequency of each overlapping clone in the dLN CD44^hi^ (left) or the tumor (right). All figures present the data for TCRβ. Two-sided unpaired Student's *t*-tests (*n* = 5). ^**^P < 0.01; ^***^P < 0.001.

Since we did not observe significant differences in the tumor repertoire between the control and aCD4 groups by analyses of the tumor compartment alone, we focused on the overlapping clones between dLN CD44^hi^ and tumor (dLN-tumor overlap), which are thought to represent CD8^+^ T cells actively participating in the Cancer-Immunity Cycle. In the TCRβ repertoire, the frequencies of the top 20 clones and the combined frequency of the dLN-tumor overlap in individual mice are depicted (Figure 4A). The total number and combined frequency of the overlapping clones in the dLN CD44^hi^ and the tumor was significantly increased in the aCD4 group (Figure 4B; total number; 82 ± 18 vs. 413 ± 43, *P* < 0.0001, combined frequency in dLN CD44^hi^; 3.4 ± 0.9% vs. 24.7 ± 1.4%, *P* < 0.0001, combined frequency in tumor; 28.8 ± 8.9% vs. 73.1 ± 5.2%, *P* = 0.0027). In the aCD4 group, the distribution plot of the top 200 overlapping clones was shifted to the upper-right in both the dLN CD44^hi^ and the tumor (Figure 4C). This tendency indicated an increase in the diversity of dLN-tumor overlap and the frequency of each overlapping clone. Hence, anti-CD4 mAb treatment is associated with an increase in dLN-tumor overlap of the CD8^+^ T cell repertoire. Similar but less clear trend was observed for the TCRα repertoire (Supplementary Figures 5A,B). Importantly, the CD44^hi^ CD8^+^ T cell repertoire in dLN contained a higher proportion of tumor-overlapping clones than whole dLN CD8^+^ T cells (Supplementary Figures 5C,D), suggesting the validity of analyzing the CD44^hi^ population of dLN CD8^+^ T cells rather than whole dLN CD8^+^ T cells.

### Anti-CD4 mAb Treatment Increased the Proportion of “dLN^major^” Clones in dLN-Tumor Overlapping Clones

**Figure 5:**
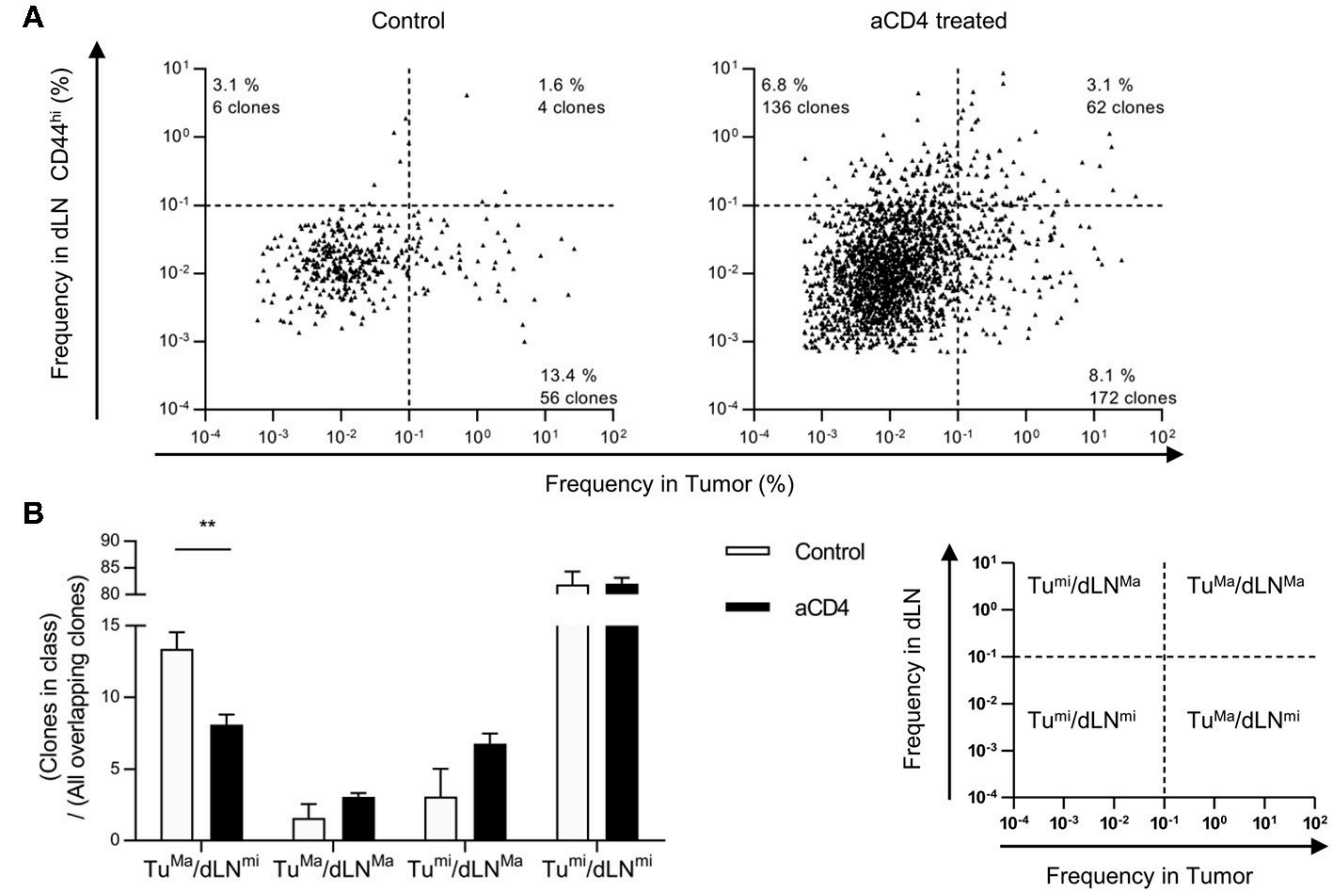
Anti-CD4 mAb treatment increased the proportion of “dLN^major^” clones in the dLN-tumor overlap. Mice bearing B16F10 tumors were injected i.p. with anti-CD4 mAb on days 5 and 9, and analyzed on day 14 after tumor inoculation. **(A)** Scatter Plot of overlapping clones between the tumor and dLN in TCRβ repertoire. Each point on the plot represents a single clone with log_10_ frequency in the tumor (x-axis) and the dLN CD44^hi^ (y-axis). The threshold for major/minor clones was defined as a frequency of 0.1% in each compartment. Plots of control (*n* = 5, left) or aCD4 (*n* = 5, right) mice were overlaid in each group. **(B)** Percentages of each pattern in overlapping clones. The percentage of each pattern was calculated in individual mice, and averaged in control and aCD4 groups. All figures present the data for TCRβ. Two-sided unpaired Student's *t*-tests (*n* = 5). ^**^P < 0.01.

To further focus on the site of enrichment of each CD8^+^ T cell clone following anti-CD4 mAb treatment, we analyzed the frequency of each overlapping clone in the tumor or dLN CD44^hi^. In the TCRβ repertoire, overlapping clones were depicted with log_10_ frequency in the tumor (x axis) and the dLN CD44^hi^ (y axis) (Scatter plot, Figure 5A). We defined “major clones” as clones with a frequency of ≥0.1% and “minor clones” as clones with a frequency of < 0.1% in each compartment. Based on this definition, we categorized overlapping clones into four patterns by their frequencies in the tumor and the dLN CD44^hi^: tumor^major^/dLN^major^, tumor^major^/dLN^minor^, tumor^minor^/dLN^major^, and tumor^minor^/dLN^minor^. We then compared the average percentages of each pattern of overlapping clones in the control and aCD4 groups (Figure 5B). In aCD4 group, the percentage of the tumor^major^/dLN^minor^ pattern decreased (control; 13.4 ± 1.2%, aCD4; 8.1 ± 0.7%, *P* = 0.0043), whereas that of the tumor^major^/dLN^major^ and tumor^minor^/dLN^major^ classes tended to increase (tumor^major^/dLN^major^: control; 1.6 ± 1.0%, aCD4; 3.1 ± 0.3%, *P* = 0.185, tumor^minor^/dLN^major^: control; 3.1 ± 1.9%, aCD4; 6.8 ± 0.7%, *P* = 0.107). These results implied that the anti-CD4 mAb treatment enhanced the enrichment of tumor-infiltrating clones in dLN.

### Patterns in dLN-Tumor Overlap Differed in Tumor Reactive Clones

**Figure 6:**
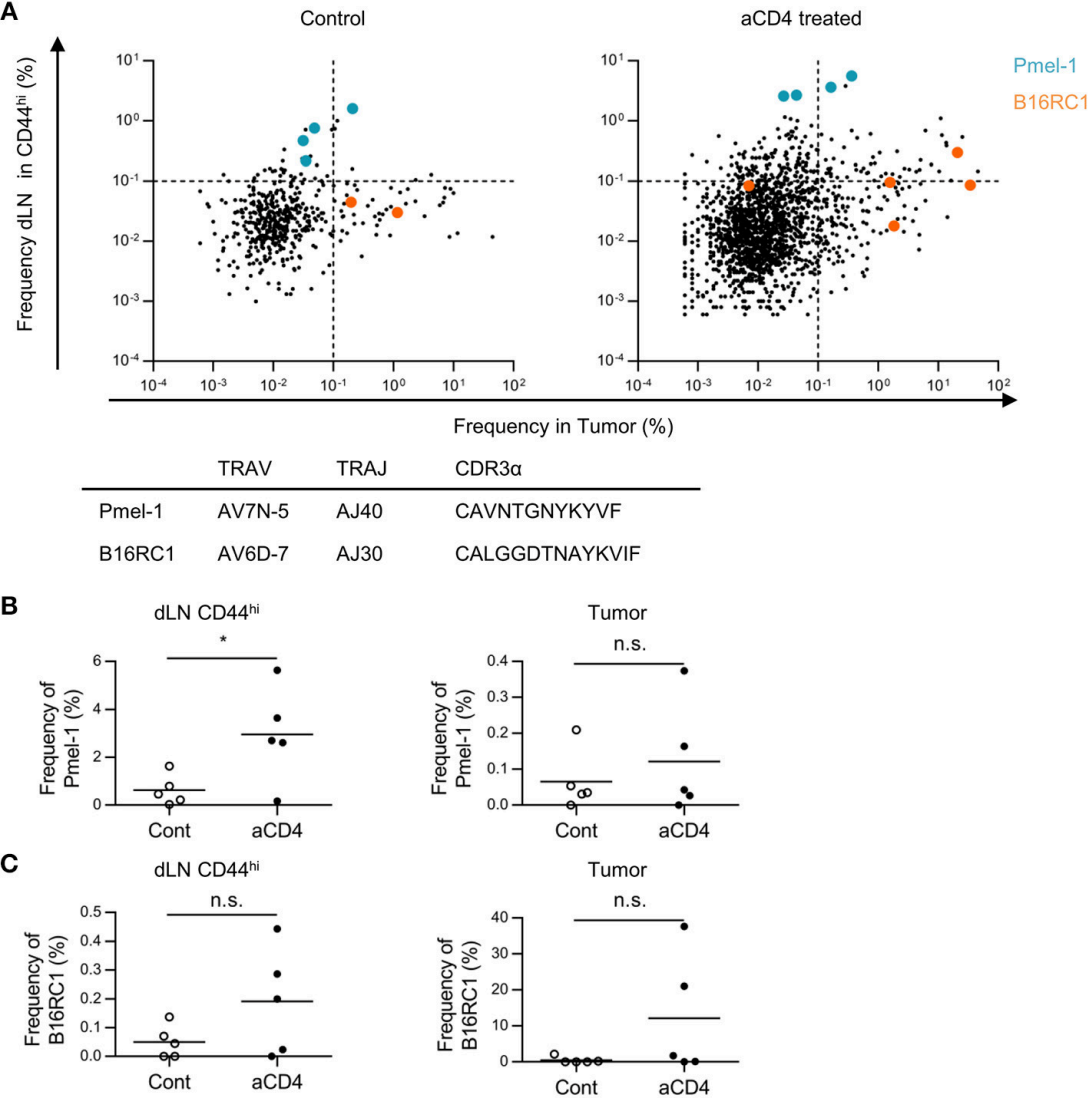
Patterns in dLN-tumor overlap differed in tumor reactive clones. Mice bearing B16F10 tumors were injected i.p. with anti-CD4 mAb on days 5 and 9, and analyzed on day 14 after tumor inoculation. **(A)** Scatter Plot of overlapping clones between the tumor and dLN in TCRα repertoire. Each point on the plot represents a single clone with log_10_ frequency in the tumor (x-axis) and the dLN CD44^hi^ (y-axis). Pmel-1 and B16RC1 are plotted in blue and orange, respectively. **(B,C)** Frequency of Pmel-1 **(B)** and B16RC1 **(C)** in dLN CD44^hi^ (left) and tumor (right). All figures present the data for TCRα. In **(B,C)**, T cell clones were determined as the TCR reads with the same TCR V segment, J segment, and CDR3 amino acid sequence. Two-sided unpaired Student's *t*-tests (*n* = 5, except for PBL of aCD4: *n* = 3). ^*^P < 0.05.

Next, we examined whether these patterns in dLN-tumor overlap differed among tumor reactive clones. To this end, we focused on two tumor-reactive CD8^+^ T cell clones; gp100 melanoma antigen-specific Pmel-1 (22) and B16F10 Reactive Clone 1 (B16RC1), which was originally cloned from CD137^high^ CD8^+^ TILs in B16F10 tumor model (23). By depicting Pmel-1 and B16RC1 in the scatter plot of dLN-tumor overlapping clones of TCRα repertoire, we found that Pmel-1 and B16RC1 had different patterns in dLN-tumor overlap; Pmel-1 was likely to be categorized into “dLN^major^” pattern, while B16RC1 was likely to be categorized into “tumor^major^” pattern (Figure 6A). Moreover, in aCD4 group, the frequency of Pmel-1 clones increased in dLN CD44^hi^ but not in tumor (dLN: control; 0.6 ± 0.3%, aCD4; 3.0 ± 0.9%, *P* = 0.037, tumor: control; 0.07 ± 0.04%, aCD4; 0.12 ± 0.07%, *P* = 0.50), whereas the frequency of B16RC1 clones showed the tendency to increase both in the dLN CD44^hi^ and tumor (dLN: control; 0.05 ± 0.03%, aCD4; 0.19 ± 0.08%, *P* = 0.14, tumor: control; 0.49 ± 0.41%, aCD4; 12.1 ± 7.5%, *P* = 0.16) (Figures 6B,C). Therefore, although Pmel-1 and B16RC1 were tumor reactive and increased following aCD4 mAb treatment, these two clones had different patterns in dLN-tumor overlap.

### Anti-CD4 mAb Treatment Enhanced the Translocation of Tumor-Infiltrating Clones Among the dLN, PBL, and Tumor

**Figure 7:**
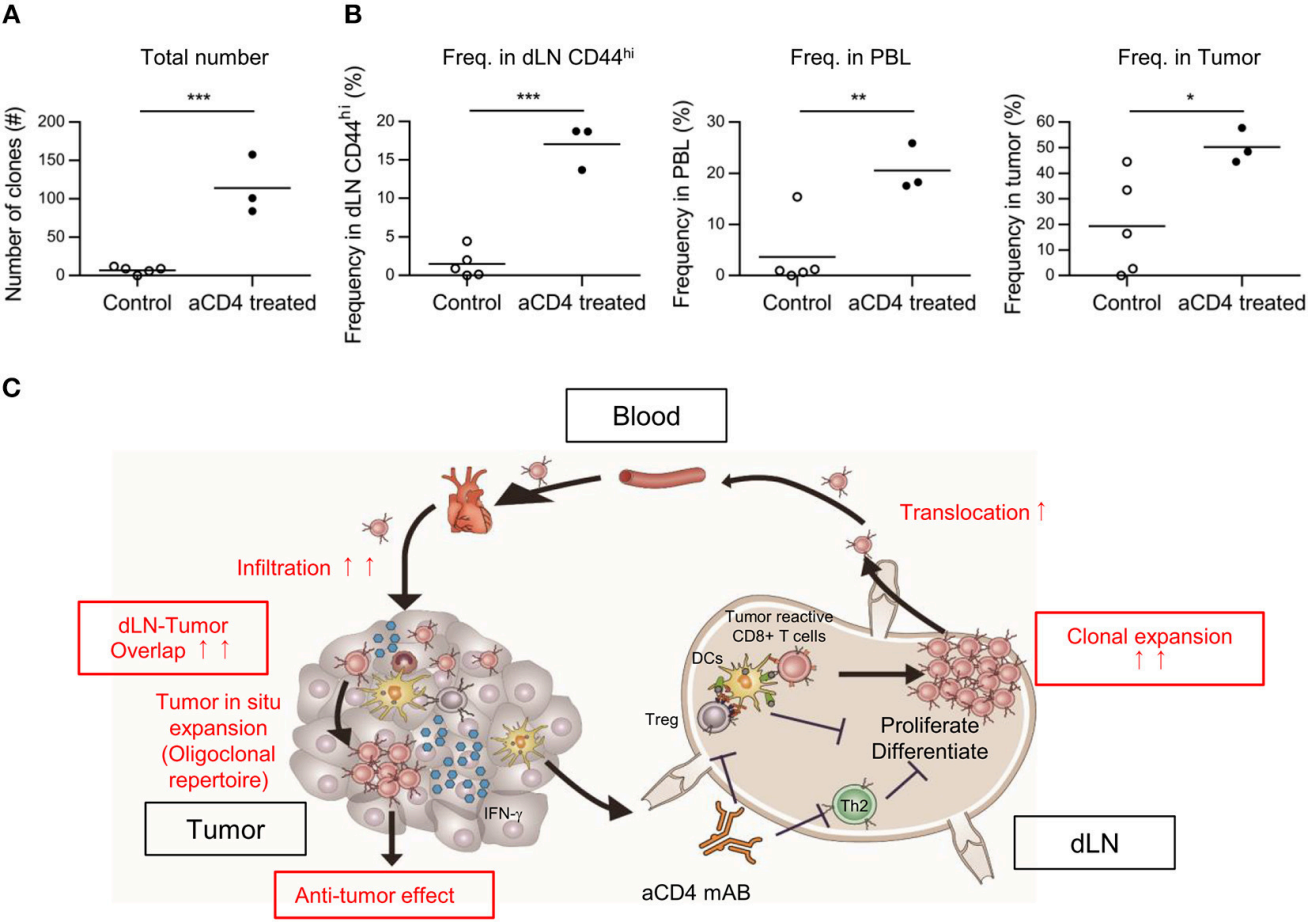
Anti-CD4 mAb treatment enhanced the translocation of tumor infiltrating clones between the dLN, the PBL, and the tumor. Mice bearing B16F10 tumors were injected i.p. with anti-CD4 mAb on days 5 and 9, and analyzed on day 14 after tumor inoculation. **(A,B)** Overlapping clones among the dLN CD44^hi^, PBL, and tumor, which were putative tumor-reactive clones. The total number **(A)** and combined frequency in the dLN CD44^hi^ (**B**, left), PBL (**B**, middle), and tumor (**B**, right) are compared. **(C)** Schema of the hypothesis for the mechanism underlying the antitumor effect of anti-CD4 mAb treatment based on TCR repertoire analysis. **(A,B)** present the data for TCR. Two-sided unpaired Student's *t*-tests (*n* = 3 for aCD4, and *n* = 5 for control). ^*^P < 0.05; ^**^P < 0.01; ^***^P < 0.001.

Based on the Cancer-Immunity Cycle, tumor reactive clones primed in dLN translocate to the tumor via blood circulation. Therefore, if the dLN-tumor overlapping repertoire increases, the dLN-PBL and PBL-tumor overlapping is also thought to increase. To probe this, we analyzed the dLN-PBL-tumor overlapping clones and compared them between the control and the aCD4 groups. In the TCRβ repertoire, the count of dLN-PBL-tumor overlapping clones significantly increased in the aCD4 group (Figure 7A control; 7 ± 2, aCD4; 114 ± 22, *P* = 0.0006). The combined frequency in dLN CD44^hi^ (control; 1.5 ± 0.8%, aCD4; 17.0 ± 1.7%, *P* < 0.0001), PBL (control; 3.7 ± 2.9%, aCD4; 20.6 ± 2.7%, *P* = 0.0083), or tumor (control; 19.4 ± 8.6%, aCD4; 50.3 ± 3.9%, *P* = 0.041) was also significantly increased (Figure 7B). The TCRα repertoire showed similar tendencies, although the increase in the combined frequency in PBL was less clear (Supplementary Figures 5E,F). These results suggested that the anti-CD4 mAb treatment also enhances the translocation of tumor-reactive CD8^+^ T cells from the dLN to PBL and PBL to the tumor, and that PBL can be used as a substitute for the dLN in the IOCT analysis, at least in part.

## Discussion

In this study, we conducted high-throughput TCR-seq on CD8^+^ T cells sorted from the dLN, PBL, and tumor in a B16F10 mouse melanoma model to investigate the effects of anti-CD4 mAb treatment on the CD8^+^ T cell repertoire. By focusing on overlapping clones among tissue compartments, we revealed that a small fraction of CD8^+^ T cell repertoire in the dLN or PBL was enriched oligoclonally in the tumor, and that the anti-CD4 mAb treatment increased the dLN-PBL-tumor overlap. Moreover, we found that the dLN-tumor overlapping clones were categorized into four patterns based on their frequencies in the dLN and tumor, and that anti-CD4 mAb treatment increased the proportion of tumor^minor^/dLN^major^ and tumor^major^/dLN^major^ clones. These findings contributed to establish TCR repertoire analysis as a response marker of anti-CD4 mAb therapy for solid tumors. We are now in the process of conducting a phase I clinical trial for a humanized anti-human CD4 depleting antibody IT1208 in Japan to promote the anti-tumor immune response in humans.

An advantage of our TCR-seq in the mouse model was purification of the T cell subpopulation before TCR repertoire analysis. One limitation of TCR repertoire analyses in previous clinical studies is that they were performed on heterogeneous subpopulations containing both CD4^+^ immunosuppressive cells (i.e., Treg and Th2) and CD8^+^ T cells, due to which the interpretation of repertoire structure or treatment-induced repertoire changes is difficult. Therefore, we separated the CD8^+^ T cells from CD4^+^ cells and interpreted their repertoire as a reflection of the CTL response. In addition, our intravascular staining (IVS) technique enabled precise identification of TILs (17). Application of similar approaches to other T cell subpopulations, such as Th1, Th2, or Tregs could provide new information on the site of clonal expansion and translocation of T cell subpopulations, and might identify clones that are more likely to differentiate into specific subpopulations.

In this study, we performed repertoire analysis of overlapping clones between tissue compartments (Inter-Organ Clone Tracking analysis, IOCT). One reason for using IOCT analysis is that the extent of repertoire overlap between the dLN and tumor differed more markedly between the aCD4 and control groups. By comparing the repertoire in each tissue compartment alone, we could not observe clear differences in the tumor or in the PBL repertoire between aCD4 and control mice. On the other hand, by focusing on overlapping clones between tissue compartments, we found that, the dLN-tumor and dLN-PBL-tumor overlapping clones were significantly increased in terms of diversity and combined frequency in the aCD4 mice (Figures 4, 7). Based on these results from IOCT analysis, and the spatiotemporal response of tumor-reactive T cells in the Cancer-Immunity Cycle, we speculate that anti-CD4 mAb treatment enhanced the expansion of a broad spectrum of tumor-reactive CD8^+^ T cell clones in the dLN and their translocation to the tumor via PBL (Figure 7C). In a previous study (7), the antitumor effect of anti-CD4 mAb treatment was reported to be associated with augmented proliferation of tumor-specific CD8^+^ T cells using a TCR transgenic system; the effect of anti-CD4 mAb on the endogenous CD8^+^ T cell clones in the tumor-bearing host remained elusive. In the current study, IOCT analysis provided the possibility that clonal expansion and tumor infiltration also occurred in the endogenous CD8^+^ T cell clones.

By analyzing the dLN-tumor overlapping clones, we revealed that the major clones in the dLN and tumor did not always match. Moreover, we could categorize the dLN-tumor overlapping clones based on their frequencies in the dLN and tumor, which seemed to represent the “organ preference for expansion.” Note that “expansion” includes not only proliferation but also migration into the tissue compartment, or retention and survival within the tissue compartment, in this case. Interestingly, two B16F10 tumor reactive clones, Pmel-1 (22) and B16RC1 (23), had different tendencies in organ preference for expansion, which may represent the differences in phenotypes between these two clones. Considering that the proliferation of polyclonal CD44^hi^ CD8^+^ T cells occurs in both the dLN and tumor in a similar B16F10 subcutaneous tumor model (7), the tumor^minor^/dLN^major^ clones may proliferate greatly in the dLN but not in the tumor, whereas the tumor^major^/dLN^minor^ clones that predominate the T cell repertoire in the tumor may proliferate weakly in the dLN but greatly in the tumor. From this finding, we hypothesize that accumulation of tumor-reactive clones in the tumor is explained by two independent processes: “expansion in dLN” and “tumor *in situ* expansion.” Interestingly, anti-CD4 mAb treatment mainly increased the proportion of “dLN^major^” clones, which is consistent with our previous observation that anti-CD4 mAb treatment promotes the cell-cycle progression of CD44^hi^ CD8^+^ T cells in the dLN but not in the tumor (7).

The possibility of tumor *in situ* expansion and proliferation of T cells is also proposed in a study on the human TCR repertoire analysis in breast cancer (24) and pancreatic ductal adenocarcinoma (25). However, the immunological basis explaining the difference between tumor^minor^/dLN^major^ and tumor^major^/dLN^minor^ categories are unknown. Thus, the antigen specificity and underlying mechanisms defining the organ preference for the expansion of T cell clones should be analyzed using single-cell TCR-seq and subsequent functional analyses (26, 27). In any case, we believe that the organ preference for the expansion of T cell clones would be an important factor for designing TCR-T cell therapy; i.e., the tumor^major^/dLN^major^ clones seem to be more favorable for transfer into cancer patients.

Despite several advantages in our experimental system, there are some limitations to our TCR repertoire analysis. First, we do not have any information about the antigen-specificity of each clone. Several groups have attempted to cluster TCR clones based on the similarity between their CDR3 sequences, which seems to represent their specific antigens (28, 29). Our TCR-seq data may also be interpreted from the viewpoint of their antigens using these methods. Second, longitudinal analysis of the TCR repertoire is very difficult in mice due to a small blood volume. In addition, the ratio of naïve/memory T cell populations in specific pathogen-free young mice is considerably different from that in aged human individuals, which limits the extrapolation of our results to the CD8^+^ T cell repertoire in cancer patients. Finally, the difficulty in sampling the tumor and lymph nodes from human cancer patients should also be considered in the development of TCR repertoire analysis as an immune monitoring system.

In conclusion, we performed TCR sequencing and IOCT analysis on anti-CD4 mAb treated tumor-bearing mice and demonstrated that anti-CD4 mAb treatment enhanced the expansion of a wide variety of CD8^+^ T cell clones in the dLN and their translocation to the tumor. We expect that the experimental system and analysis module used in this study might reveal the effect of other immune checkpoint inhibitors toward the spatiotemporal response of T cell clones in the Cancer-Immunity Cycle, and contribute to establish the TCR repertoire as an important response marker in cancer immunotherapy.

## Data Availability Statement

The datasets generated for this study can be found in the NCBI GEO; accession GSE115425.

## Author Contributions

HA and SU wrote the manuscript, designed the concepts, analyzed data, performed research, and revised the manuscript. SS developed methodology, performed research, and revised the manuscript. HO performed research. SH developed methodology and revised the manuscript. KK, SI, and KM designed the concepts and revised the manuscript.

### Conflict of Interest Statement

SU and KM have ownership interest (including patents) in IDAC Theranostics. KM reports receiving a commercial research grant, and is a consultant/advisory board member for IDAC Theranostics. The remaining authors declare that the research was conducted in the absence of any commercial or financial relationships that could be construed as a potential conflict of interest.
