# Supplementary material for: TCR Repertoire Analysis Reveals Mobilization of Novel CD8+ T Cell Clones Into the Cancer-Immunity Cycle Following Anti-CD4 Antibody Administration
Source: Front Immunol. 2019 Jan 24;9:3185. doi: 10.3389/fimmu.2018.03185 (PMC6353793; doi:10.3389/fimmu.2018.03185)
Supplement: Supplementary file 4 [file Table_4.docx]

Supplementary Material

TCR repertoire analysis reveals mobilization of novel CD8+ T cell clones into the Cancer-Immunity Cycle following anti-CD4 antibody administration

Hiroyasu Aoki^1,2^, Satoshi Ueha^1,2*^, Shigeyuki Shichino^1,2^, Haru Ogiwara^1,2^, Shin-ichi Hashimoto^1,2,3^, Kazuhiro Kakimi^4^, Satoru Ito^1,2,5^, Kouji Matsushima^1,2^

*** Correspondence:** Dr. Satoshi Ueha: ueha@rs.tus.ac.jp

## 1. Supplementary Methods

**High-throughput sequencing of the TCR repertoire**

TCR sequencing libraries for next generation sequencing were prepared from the mRNA of sorted CD8^+^ T cell samples according to the protocol described in (Shichino et al. *JCI Insight*, 2018, in press, GSE110711) with some modifications. Briefly, 5 pmol of BioEcoP-dT25-adapter was bound to 20 μL of Dynabeads M270 streptavidin (Thermo Fisher Scientific, MA, USA, #DB65305). The washed beads (20 uL) were added to each cell lysis buffer [1% LiDS, 100 mM Tris-HCl (pH 7.5), 500 mM LiCl, 10 mM EDTA, and 5 mM DTT] and incubated for 30 min at room temperature with gentle rotation. Beads were washed once with wash buffer A [0.1% LiDS, 10 mM Tris-HCl (pH 7.5), 150 mM LiCl, and 1 mM EDTA] and three times with wash buffer B [10 mM Tris-HCl (pH 7.5), 150 mM LiCl, and 1 mM EDTA]. Beads were then suspended in 10 μL of RT mix 1 [1× SSIV buffer (Thermo Fisher Scientific), 2 mM dNTP, 12 mM MgCl2, and 3.2 U/μL RNaseIn Plus (Promega)] and incubated for 90 s at 70ºC, 5 min at 35ºC, and immediately cooled on ice. RT mix 2 [10 μL; 1× SSIV buffer, 10 mM DTT (Thermo Fisher Scientific), 10 U/μL Superscript IV (Thermo Fisher Scientific), and 2 M betaine (Sigma-Aldrich)] was added, and reverse transcription was performed for 5 min at 35ºC and 15 min at 50ºC. Beads were washed once with cell lysis buffer, twice with B&W-T buffer [5 mM Tris-HCl (pH 7.5), 1 M NaCl, 0.5 mM EDTA, and 0.1% Tween-20], once with Tris-HCl (pH 8.0) and added 20 μL of RNase H mix [1× first-strand buffer (Life Technologies, Carlsbad, CA, USA), 5 mM DTT, 0.6 U RNase H (Thermo Fisher Scientific)], and incubated for 20 min at 37ºC to digest reverse-transcribed mRNA. Beads were washed, 20 μL of TdT mix [50 mM Tris-HCl (pH 8.0), 100 mM KCl, 3 mM MgCl2, 1 mM CoCl2 (Roche), 0.65 mM dATP (Thermo Fisher Scientific), and 15.2 U/μL TdT (Roche)] was added on an ice-chilled aluminum rack, and polyA-tailing was performed for 3 min 20 s at 37ºC. The reaction was stopped by adding 5 μL of 0.5 M EDTA, and the enzyme was heat-inactivated by incubation for 10 min at 65ºC. Beads were washed, 25 μL of second-strand synthesis mix [1× KAPA Hifi ReadyMix (KAPA Biosystems, Wilmington, MA, USA) and 0.4 µM anchored tagging primer] was added, and second-strand synthesis was performed according to the following program: 95ºC for 2 min, 98ºC for 20 s, 44ºC for 2 min, 72ºC for 7 min, and hold at 4ºC. Beads were washed, and 1/4 of the beads were used for the first round of whole-transcript amplification (WTA) in 25 μL of first-round WTA mix [1x NEBNext Ultra II Q5 Master Mix (New England Biolabs, MA, USA), 0.4 µM 5’ WTA primer, and 0.4 µM 3’ WTA primer] using the following program: 98ºC for 1 min, five cycles of 98ºC for 20 s, and 65ºC for 7min 15 s, followed by 72ºC for 5 min and a hold at 4ºC. PCR products were purified twice with 0.6x AmPure XP beads (Beckman Coulter, CA, USA) and eluted with 15 μL of nuclease-free water. To amplify the TCR cDNA containing complementarity determining region 3 (CDR3), nested PCR of the TCR locus was performed as follows. The first PCR mix [8 μL; 1x Q5 High-Fidelity Master Mix (New England Biolabs #M0491), 0.2 μM primer mix (5′ WTA, Trac_ex, and Trbc_ex)] was added into 2 μL of elutant, and first PCR was performed using the following program: 98°C for 30 s, 10 cycles of 98 ºC for 10 s, and 65 ºC for 75 s, followed by 65 ºC for 5 min. Next, PCR products were purified twice with 0.7x AmPure XP beads (Beckman Coulter) and eluted with 10 μL of nuclease-free water. The second PCR mix [21 μL; 1x Q5 High-Fidelity Master Mix (New England Biolabs), 0.5 μM primer mix (5′ WTA, Trac_in-Bio and Trbc_in-Bio)] was added into 4 μL of elutant, and second PCR was performed using the following program: 98°C for 30 s, 18-23 cycles of 98 ºC for 10 s, and 65 ºC for 75 s, followed by 65 ºC for 5 min. Next, PCR products were purified twice with 0.7x AmPure XP beads (Beckman Coulter) and eluted with 16 μL of nuclease-free water. The purified PCR products were sheared randomly using NEBNext dsDNA fragmentase (New England Biolabs, #M0348). The fragmentation reaction mix consisted of 2.3 μL of nuclease-free water, 0.2 μL of 10 mg/ml BSA, 1 μL of 100 mM MgCl_2_, 2 μL of 10x reaction buffer, 12.5 μL of 8 ng/μL of PCR product, and 2 μL of Fragmentase. The fragmentation reaction was incubated at 37°C for 30 min, and then 5 μL of 0.5 M EDTA was added to stop the reaction. The sheared PCR products were then purified and subjected to size selection using the 0.8x AmPure XP beads (Bexkman-Coulter) to remove large fragments, and 0.8x AmPure XP beads to remove the smaller fragments, and eluted with 20 μL of Tris-HCl (pH 8.0). To capture the TCR cDNA containing the end of Constant region, the purified PCR products were incubated with 30 μL of Dynabeads M-270 Streptavidin (Thermo Fisher Scientific) for 30 min at room temperature with gentle rotation, washed 3 times with B&W-T buffer, and once with Tris-HCl (pH 8.0), and nuclease-free water. The captured TCR cDNA was repaired using NEBNext Ultra II End Repair/ dA-Tailing Module (New England Biolabs, # E7546L). The repair reaction mix contained 1.2 μL of reaction buffer, 8.3 μL of beads re-suspended with nuclease-free water, and 0.5 μL of enzyme mix. The repair reaction was incubated at 20°C for 30 min, then washed once with lysis buffer, 3 times with B&W-T buffer, and once with Tris-HCl (pH 8.0). The repaired TCR cDNA was attached to the sequencing adaptor using the DNA Ligation Kit ＜Mighty Mix＞ (TaKaRa, Shiga, Japan, #6023). The ligation reaction mix consisted of 1 μL of 10 μM P1 adaptor, 6.5 μL of beads re-suspended in Tris-HCl (pH 8.0), and 15 μL of enzyme mix. The ligation reaction was incubated at 16°C for 60 min using a thermal cycler with the cover open, washed once with lysis buffer, 3 times with B&W-T buffer, and once with Tris-HCl (pH 8.0). The third PCR mix [21 μL; 1x Q5 High-Fidelity Master Mix (New England Biolabs), 0.5 µM IonA-BC[N]-Trac or Trbc-primer, and 0.5 µM trP1 primer] was added into 4 μL of resuspended beads, and PCR enrichment was performed using the following program: 98°C for 30 s, 9 cycles of 98 ºC for 10 s, and 65 ºC for 75 s, followed by 65 ºC for 5 min. The PCR products were then purified and subjected to size selection using the 0.75x AmPure XP beads (Bexkman-Coulter) to remove large fragments, and 0.65x for TCRα, or 0.5x for TCRβ, AmPure XP beads to remove the smaller fragments, and eluted with 20 μL of Tris-HCl (pH 8.0).Amplified TCR libraries were quantified using a KAPA Library Quantification Kit (KAPA Biosystems, #KK4827) and size distribution was analyzed by agarose electrophoresis and SYBR Gold staining (Thermo Fisher Scientific, #S11494). Final TCR libraries, whose lengths were 200–300 base pairs, were pooled and sequenced using an Ion Hi-Q Chef kit, an Ion PI v3 Chip kit, and an Ion Proton Sequencer (Thermo Fisher Scientific, # A27198, #A26771, # 4476610) according to the manufacturer’s instructions, except that the input library concentration was 100 pM. The raw data from these experiments have been deposited at NCBI GEO; accession GSE115425. The sequences of the primers and adapters are shown in Supplementary Table S1.

**2.** **Supplementary Figures**

**Supplementary Figure 1**

Overview of the TCR-seq method in this paper. **(A)** **1.** Total mRNA was captured using BioEcoP-dT25-adapter-bound streptavidin magnetic beads. **2.** Captured mRNA was converted to cDNA using reverse transcriptase. **3.** Remaining mRNA was digested and poly A tail was added to 3’ end of cDNA. **4.** Primer containing poly T and universal sequence (5’ WTA) was annealed to poly A tail of cDNA, and 2^nd^ strand was synthesized by PCR. **5.** TCR locus containing CDR3 and V region were amplified by 3step nested PCR. **6.** Amplified TCR cDNA was fragmented enzymatically. **7.** TCR fragment containing biotinylated C region were captured by streptavidin magnetic beads. **8.** The captured TCR cDNA was end-repaired, dA-tailed, and ligated with adaptor sequence (P1 adaptor). **9.** The sequencing adaptor and barcode were then added using PCR. **(B)** Electropherogram of the final TCR library. The length of the final TCR library for Ion Proton next generation sequencing was about 200-300 base pairs.

**Supplementary Figure 2**

Accuracy and reproducibility of the TCR-seq method. **(A)** Accuracy of the TCR-seq method. Correlation of frequency between the Pmel-1 TCR sequence in NGS reads and Pmel-1 CD8^+^ T cells in flow cytometry plots was calculated for TCRα (left) and TCRβ (right). The correlation coefficient was calculated as the Pearson’s product-moment correlation coefficient. **(B,C)** Reproducibility of the TCR-seq method. Overlapping clones between technical replicate samples were plotted with log_10_ frequencies in each sample. The correlation coefficient was calculated as the Pearson product-moment correlation coefficient **(B)**. Frequency of overlapping clones between replicate samples are shown for the top 20 overlapping clones, as well as the clones whose frequency were below the top 20 (black) and non-overlapping (grey) clones, and the cumulative frequency of overlapping clones is calculated and shown at the top of the graph **(C)**. This experiment was done in duplicate. **(B,C)** present the data for TCRβ.

**Supplementary Figure 3**

Differences in TCRα repertoires between the dLN, PBL, and tumor in tumor-bearing mice. Mice bearing B16F10 tumors were analyzed on day 14 after tumor inoculation. **(A)** V/J segment usage plots of the dLN CD44^hi^, PBL, and tumor repertoires of a control mouse. Ribbons connecting the V and J segments are scaled by the corresponding V/J pair frequency. **(B)** Quantile statistics of the dLN CD44^hi^, PBL, and tumor in a control mouse. The second layer of the pie chart displays the combined frequency of the top 20%, 20–40%, 40–60%, 60–80%, and 80–100% clones. The third layer displays the individual frequency of the top 5 clones. **(A,B)** are figures depicting data from the same mouse. **(C)** Average of quantile statistics in control mice (n = 5). **(D)** IOCT analysis in individual control mice. The top 100 overlapping clones in at least two compartments are colored by the tissue compartment in which their frequency was the highest, whereas the clones whose frequency were below the top 100 are shown as light gray and labelled “Collapsed” in the figure legend*.* **(E)** Variability of TCR repertoire between individual mice in the dLN CD44^hi^, PBL, and tumor. The top 10 clones of each individual mice were plotted on the heat map. Each column and row represent the individual mice and clones, respectively. Colors represent the log10 scaled frequency of clones in each mouse as indicated by a color scale. Undetected clones are colored white. **(A-C)** present the data of control group. In **(E)**, T cell clones were determined as the TCR reads with the same TCR V segment, J segment, and CDR3 amino acid sequence. All figures present the data for TCRα.

**Supplementary Figure 4**

The comparison of the TCRα repertoire in the dLN, PBL, and tumor between control and aCD4 groups. Mice bearing B16F10 tumors were injected i.p. with anti-CD4 mAb on days 5 and 9, and were analyzed on day 14 after tumor inoculation. **(A)** Quantile statistics of the dLN, dLN CD44^hi^, PBL, and tumor repertoire. **(B)** Distribution plots of the dLN, dLN CD44^hi^, PBL, and tumor repertoire. The x-axis represents each clone in the descending order of frequency, and y-axis represents the frequency of each clone in the dLN, dLN CD44^hi^, PBL, and tumor repertoires. **(C,D)** Clonality **(C)** and diversity **(D)** of the dLN, dLN CD44^hi^, PBL and tumor repertoire. Clonality was calculated as 1 - Pielou index (method). Diversity was defined as the number of clones with a frequency of > 0.01%. All figures present the data for TCRα. Two-sided unpaired Student’s *t*-tests (n = 5, except for dLN of control: n = 4 and PBL of aCD4: n = 3). *, P < 0.05; **, P < 0.01; ***, P < 0.001.

**Supplementary Figure 5**

The IOCT analysis on TCRα repertoire. Mice bearing B16F10 tumors were injected i.p. with anti-CD4 mAb on days 5 and 9, and analyzed on day 14 after tumor inoculation. **(A)** Overlapping clones between the dLN CD44^hi^ and tumor. The total number (left), combined frequency in the dLN CD44^hi^ (middle), and combined frequency in the tumor (right) were compared. **(B)** Distribution plots of overlapping clones between the dLN CD44^hi^ and tumor. The x-axis represents each overlapping clone in the descending order of frequency, and the y-axis represents the frequency of each overlapping clone in the dLN CD44^hi^ (left) or the tumor (right). **(C,D)** Overlapping clones between the dLN and tumor. Repertoire overlap between the tumor and whole CD8^+^ or CD44^hi^ CD8^+^ T cells in the dLN was compared in terms of the total number of clones **(C)** and combined frequency in dLN **(D)**. **(E,F)** Overlapping clones among the dLN CD44^hi^, PBL, and tumor, which were putative tumor-reactive clones. The total number **(E)**, and combined frequency in the dLN CD44^hi^ (**F**, left), PBL (**F**, middle), and tumor (**F**, right) are compared. All figures present the data for TCRα. Two-sided unpaired Student’s *t*-tests (n = 5 for aCD4, and n = 4 for control). *, P < 0.05; **, P < 0.01; ***, P < 0.001.
