# Supplementary material for: TCR Repertoire Analysis Reveals Mobilization of Novel CD8+ T Cell Clones Into the Cancer-Immunity Cycle Following Anti-CD4 Antibody Administration
Source: Front Immunol. 2019 Jan 24;9:3185. doi: 10.3389/fimmu.2018.03185 (PMC6353793; doi:10.3389/fimmu.2018.03185)
Supplement: Supplementary file 5 [file Presentation_1.PPTX]

## Slide 1
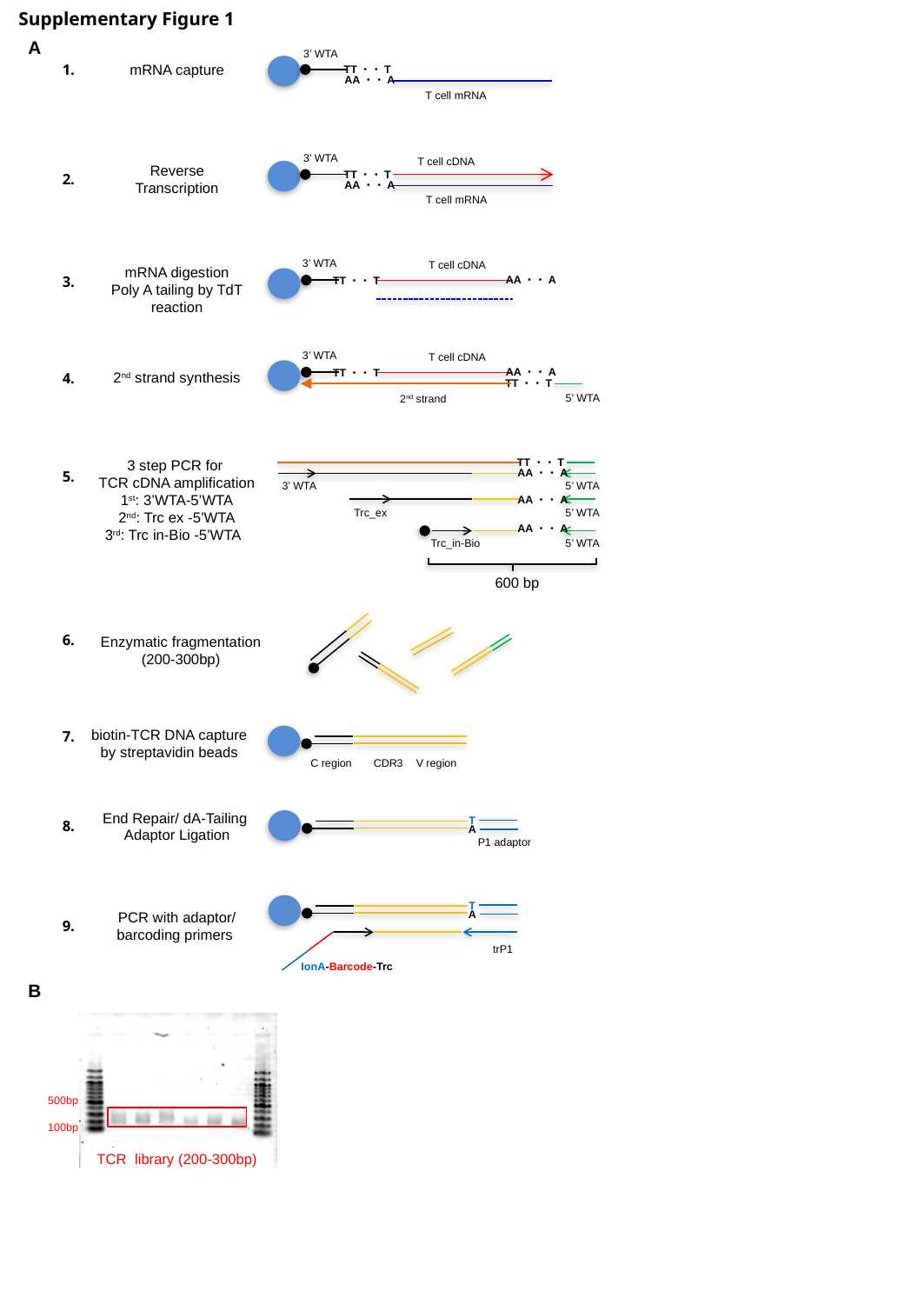

Supplementary Figure 1
A
3’ WTA
TT・・T
AA・・A
T cell mRNA
1.
mRNA capture
3’ WTA
TT・・T
AA・・A
T cell mRNA
T cell cDNA
Reverse Transcription
2.
3’ WTA
TT・・T
T cell cDNA
AA・・A
mRNA digestion
Poly A tailing by TdT reaction
3.
3’ WTA
TT・・T
T cell cDNA
AA・・A
2nd strand synthesis
4.
TT・・T
5’ WTA
2nd strand
TT・・T
3 step PCR for
TCR cDNA amplification
1st: 3’WTA-5’WTA
2nd: Trc ex -5’WTA
3rd: Trc in-Bio -5’WTA
AA・・A
5.
3’ WTA
5’ WTA
AA・・A
Trc_ex
5’ WTA
AA・・A
Trc_in-Bio
5’ WTA
600 bp
6.
Enzymatic fragmentation
(200-300bp)
biotin-TCR DNA capture by streptavidin beads
7.
C region
CDR3
V region
End Repair/ dA-Tailing
Adaptor Ligation
T
8.
A
P1 adaptor
T
A
PCR with adaptor/ barcoding primers
9.
trP1
IonA-Barcode-Trc
B
500bp
100bp
TCR library (200-300bp)

## Slide 2
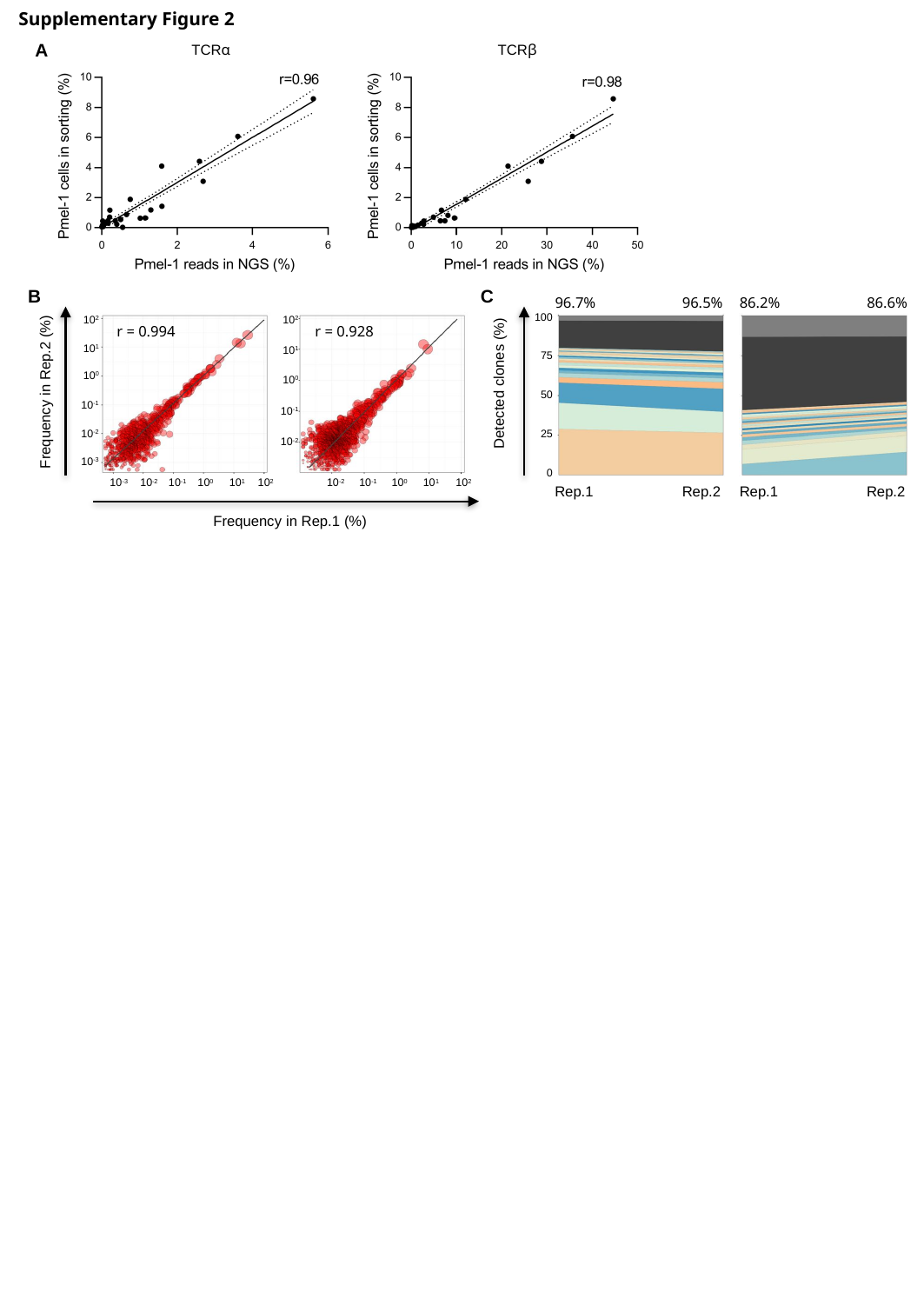

Supplementary Figure 2
A
TCRα
TCRβ
B
C
96.7%
96.5%
86.2%
86.6%
100
75
50
25
0
102
101
100
10-1
10-2
102
101
100
10-1
10-2
10-3
r = 0.994
r = 0.928
Detected clones (%)
Frequency in Rep.2 (%)
10-3
10-2
10-1
100
101
102
10-2
10-1
100
101
102
Rep.1
Rep.2
Rep.1
Rep.2
Frequency in Rep.1 (%)

## Slide 3
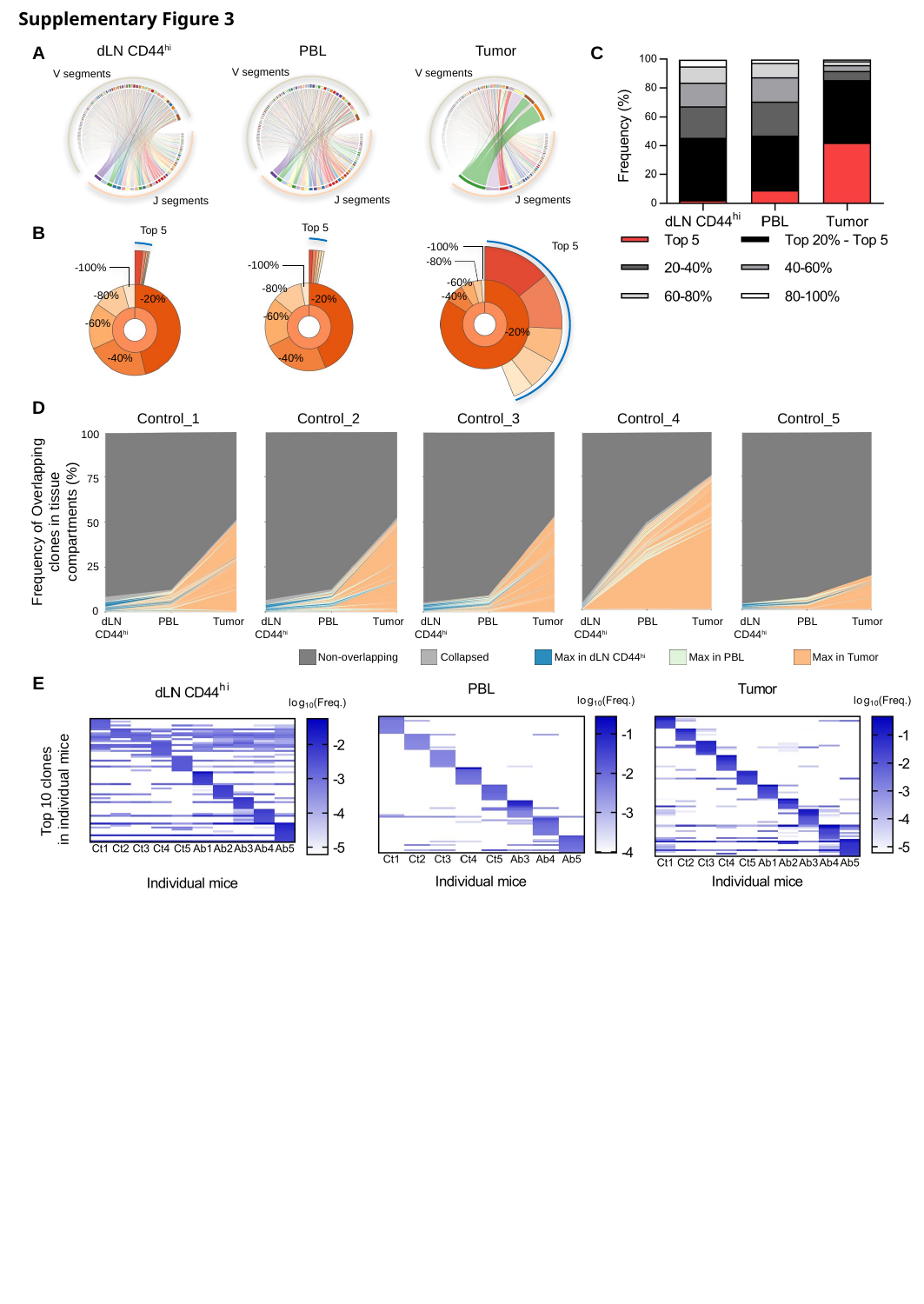

Supplementary Figure 3
dLN CD44hi
PBL
Tumor
C
A
V segments
J segments
V segments
J segments
V segments
J segments
Top 5
-100%
-80%
-20%
-60%
-40%
B
Top 5
Top 5
-100%
-100%
-80%
-60%
-40%
-80%
-60%
-40%
-20%
-20%
D
Control_4
Control_5
Control_1
Control_2
Control_3
100
75
50
25
0
Frequency of Overlapping clones in tissue compartments (%)
dLN CD44hi
PBL
Tumor
dLN CD44hi
PBL
Tumor
dLN CD44hi
PBL
Tumor
dLN CD44hi
PBL
Tumor
dLN CD44hi
PBL
Tumor
Non-overlapping
Collapsed
Max in dLN CD44hi
Max in PBL
Max in Tumor
E
Top 10 clones
in individual mice

## Slide 4
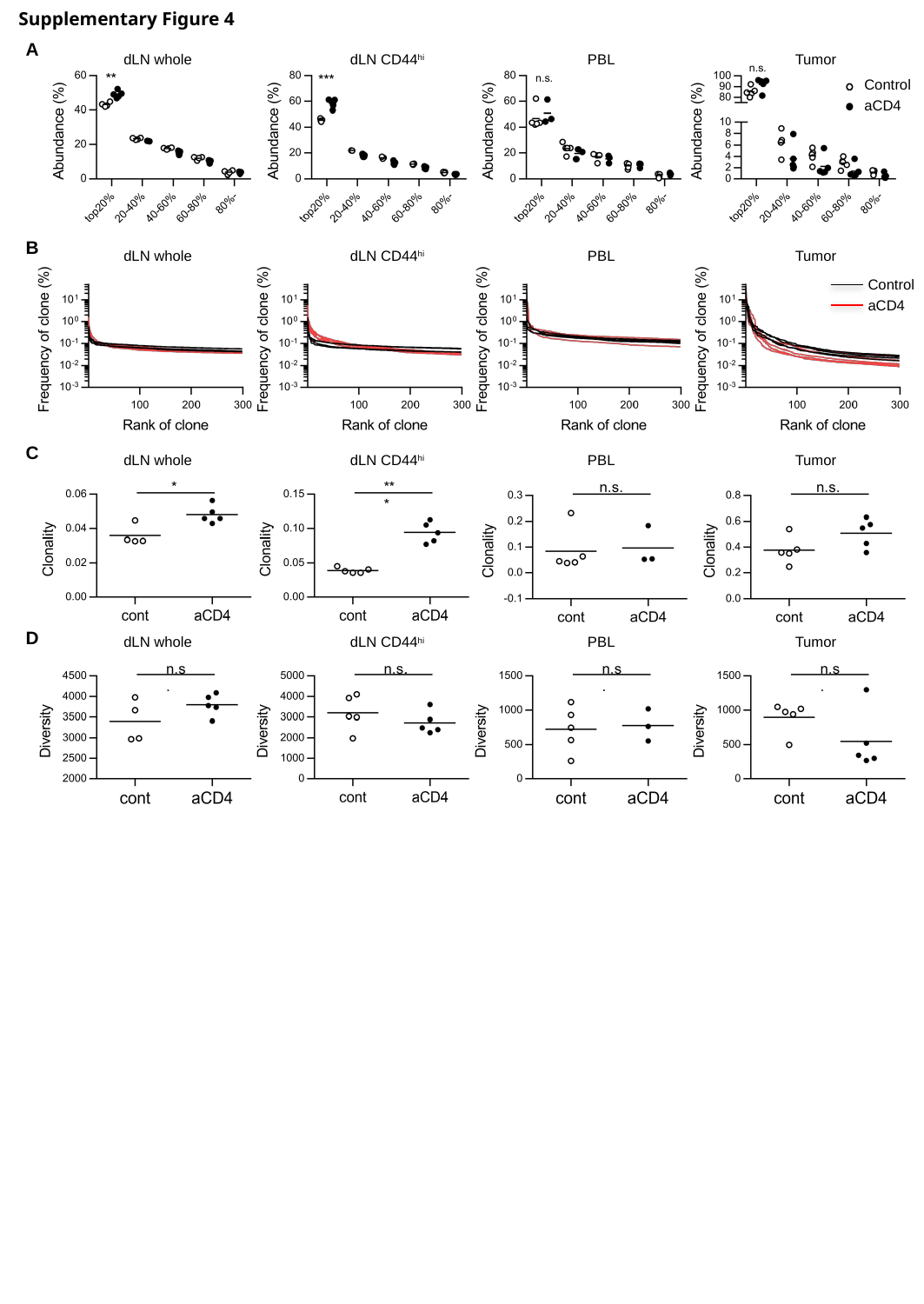

Supplementary Figure 4
A
dLN whole
dLN CD44hi
PBL
Tumor
Control
aCD4
B
dLN whole
dLN CD44hi
PBL
Tumor
Control
aCD4
C
dLN whole
dLN CD44hi
PBL
Tumor
D
dLN whole
dLN CD44hi
PBL
Tumor

## Slide 5
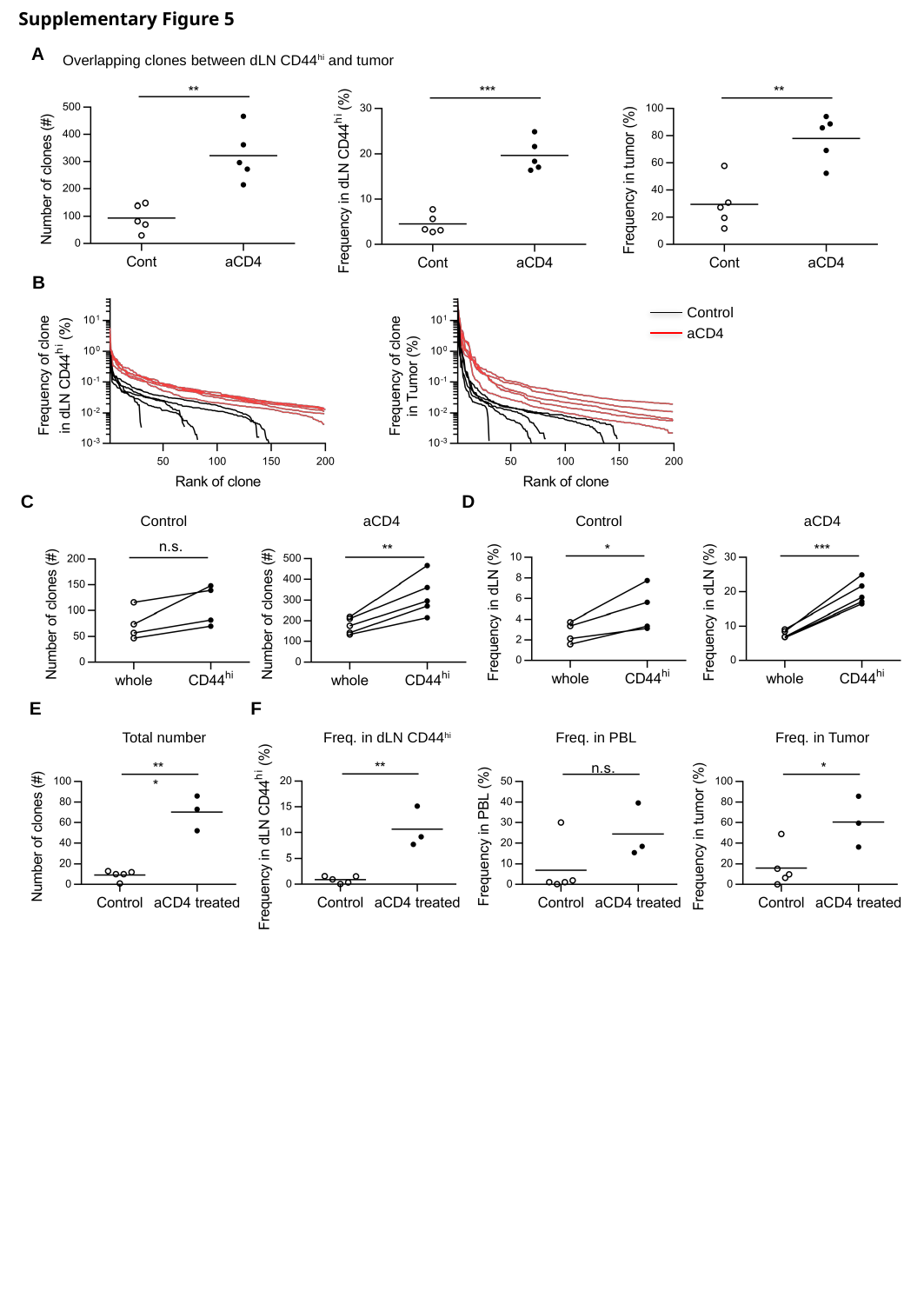

Supplementary Figure 5
A
Overlapping clones between dLN CD44hi and tumor
B
Control
aCD4
C
D
Control
aCD4
Control
aCD4
E
F
Total number
Freq. in dLN CD44hi
Freq. in PBL
Freq. in Tumor
